# Effect of antiplatelet and anticoagulant medications on implant survival: a long-term retrospective cohort study

**DOI:** 10.1007/s10006-025-01341-7

**Published:** 2025-01-23

**Authors:** Georgios S. Chatzopoulos, Larry F. Wolff

**Affiliations:** 1 Department of Developmental and Surgical Sciences, Division of Periodontology, School of Dentistry, University of Minnesota, 515 Delaware Street SE, Minneapolis, MN 55455 USA; 2 Department of Preventive Dentistry, Periodontology and Implant Biology, School of Dentistry, Aristotle University of Thessaloniki, Thessaloniki, 54124 Greece

**Keywords:** Anticoagulants, Antiplatelets, Dental implants, Survival, Retrospective

## Abstract

**Purpose:**

This large-scale retrospective study aimed to examine the long-term effect of antiplatelet and anticoagulant medications intake on dental implant treatment outcome.

**Materials and methods:**

This study retrospectively examined data from patients who underwent dental implant procedures at several university dental clinics within the BigMouth network between 2011 and 2022. Patients’ characteristics including age, gender, ethnicity, race, tobacco use, systemic medical conditions and intake of antiplatelets and anticoagulants were analyzed. Implant treatment outcome was the main outcome variable. Implant failure was defined as the removal of a dental implant for any reason. Time to failure (date of procedure to date of visit with failure) was recorded, while sites without a failure were censored at the last follow-up visit.

**Results:**

A total of 50,333 dental implants in 20,842 patients over 12 years were analyzed and an implant failure rate of 1.4% at the implant level and 2.7% at the patient level were found. Asians, African-Americans, American Indians or Alaskan Natives, and White individuals were significantly more likely to receive antiplatelet medications than Hispanics or Latinos. Males and smokers exhibited significantly higher odds of being antiplatelet and anticoagulant users compared to females and non-smokers, respectively. When the implant survival rates between antiplatelet and anticoagulant users were compared to non-users, no significant differences were observed.

**Conclusion:**

Within the limitations of this study, it appears that the use of anticoagulant and antiplatelet medications does not affect the risk of implant failure. Both anticoagulant and antiplatelet users and non-users exhibit similar high implant survival rates.

## Introduction

Dental implants are a widely used and successful treatment for tooth loss, but there is a risk of complications that can impact osseointegration [1]. Multiple studies have demonstrated high long-term success rates for dental implants in the general population, with survival rates ranging from 90 to 95% across diverse clinical situations and patient demographics [2–7]. Despite this overall positive outlook, specific factors can increase the risk of implant failure in some individuals, leading to lower success rates and potential implant loss. Implant failures are categorized based on when they happen: early failures occur within a year of placement, while late failures happen after the implant has been functioning for over a year [8]. Factors such as systemic diseases may also influence implant success and survival, but more research is needed to fully understand their impact.

During long-term evaluations of dental implants, two key measures are used: implant survival, which demonstrates that an implant remains in place, and implant success encompassing both the implant’s presence and the health of the surrounding hard and soft tissues [9]. It can be considered that survival reflects the implant’s ability to function, whereas success gauges its ability to function without causing any further complications. Research has highlighted a number of risk factors affecting the survival and success of dental implants. These factors include the existing systemic diseases, lifestyle habits such as smoking, medications, and the specific characteristics of the bone where the implant is placed [10–12].

The survival of dental implants depends on osseointegration. Bone metabolism, encompassing the processes of bone formation and remodeling, plays a key role in achieving successful osseointegration [13]. If bone metabolism is disrupted, it can negatively impact osseointegration and potentially result in implant failure [13]. Individuals with an increased risk of blood clot formation, such as those with or at risk for conditions like pulmonary embolism, deep vein thrombosis, atrial fibrillation, valvular transplant, ischemic heart disease, and stroke, are often prescribed anticoagulant and antiplatelet medications to help prevent these potentially life-threatening events [14].

Platelets, through their activation and aggregation, release growth factors that significantly contribute to the early stages of osseointegration [15, 16]. Therefore, clopidogrel, a medication that inhibits platelet aggregation, may indirectly impact bone healing. Furthermore, clopidogrel could potentially affect bone healing directly as bone cells have receptors (P2Y12) that clopidogrel interacts with [17–19]. The role of these receptors and the impact of blocking them with drugs like clopidogrel on bone health and healing is still being researched and controversial [19–24]. With respect to anticoagulant treatment, there are two main types: direct oral anticoagulants (DOACs) and vitamin K antagonists (VKAs). Research suggests that heparin negatively affects bone health leading to loss of bone density [25]. In contrast, low molecular weight heparins appear to be a safer option [26]. While VKAs have a significant impact on bone metabolism, specifically on osteocalcin, their effect on bone mineral density and fracture risk is still debated [27, 28]. Current evidence indicates that DOACs might have a less detrimental effect on bone metabolism compared to VKAs, and may potentially lead to a lower risk of fractures [29, 30]. However, further well-designed studies are necessary to confirm this. Additionally, it is important to recognize that not all DOACs are equal in this respect. Recent data reported that rivaroxaban and apixaban might offer advantages over VKAs in terms of bone health, while dabigatran may not [31]. Therefore, the findings regarding the influence of antiplatelet and anticoagulant medications on bone health are controversial and unclear.

Current research on the association between antiplatelets and anticoagulants with dental implant outcome is scarce. This large-scale retrospective study aimed to examine the long-term effect of antiplatelet and anticoagulant medications intake on dental implant treatment outcome. Understanding the potential impact of medications on implant outcomes is critical. The study hypothesizes that antiplatelets and anticoagulants, due to their potential negative effects on bone health, may increase the risk of dental implant failure.

## Materials and methods

This study retrospectively examined data from patients who underwent dental implant procedures at several university dental clinics within the BigMouth network between 2011 and 2022. The following universities contributed data for this study: Harvard University; University of Texas Health; The University of California, San Francisco; University of Colorado; Loma Linda University; University of Buffalo; The University of Iowa; The University of Minnesota; Tufts University. The study was reviewed by the University of Minnesota Institutional Review Board (IRB), and a waiver of informed consent was granted due to the retrospective nature of the study and the use of de-identified data (#STUDY00016865, 10/10/2022). In addition, the BigMouth Consortium for Oral Health Research and Informatics approved the investigation ensuring compliance with the Helsinki Declaration’s ethical guidelines.

The study specifically focused on adult patients with missing teeth who had undergone at least one dental implant procedure. Current Dental Terminology (CDT) codes were used to identify eligible patients from electronic health records. The first step was to identify patients who had undergone either comprehensive or periodic oral examinations (CDT D0150, D0120 or D0180). From this group, individuals with missing teeth (partially or completely missing teeth) who had received at least one dental implant were identified using CDT code D6010: surgical placement of implant body: endosteal implant. Tobacco use was defined as current and never smoker based on self-reported data.

Detailed information was extracted regarding patient demographics including age, ethnicity, race, gender, tobacco use, and the specific types of antiplatelet and anticoagulant medication intake. The examined antiplatelet medications included: Acetylsalicylic acid (Aspirin); Clopidogrel (Plavix); Prasugrel (Effient); and Ticagrelor (Brillinta). The following anticoagulants were also examined: Apixaban (Eliquis); Dabigatran (Pradaxa); Edoxaban (Lixiana); Rivaroxaban (Xarelto); and Warfarin (Coumadin). Data were extracted and validated from the electronic health records by independent data analysts at The University of Texas Health Science Center at Houston.

Implant failure was defined as the removal of a dental implant for any reason, including loss of integration with the bone, mobility, persistent pain, fracture, or substantial bone loss, as assessed at the patient’s latest follow-up visit specifically using the CDT D6010 code. On the other hand, implant survival was defined as the implant remaining in place and functioning without any need for removal at the last appointment recorded in the electronic database. The final outcome for each implant was classified as either a survival or a failure.

### Statistical analysis

Patient characteristics were summarized using means and standard deviations for continuous data, and frequencies and percentages for categorical data. Time to failure was documented, while survived implants were censored until the last follow-up. At the implant level, survival analysis was performed for the antiplatelet and anticoagulant medications and Kaplan-Meier plots were generated. The hazard ratios (HR) with 95% confidence intervals (CI) were calculated using Cox regression. At the patient level, significant variables were analyzed with Fisher’s exact test and multivariate logistic regression. Variables found to be significant in univariate analysis (*p* < 0.05) were subsequently introduced into a multivariable regression model to identify parameters associated with the use of antiplatelet and/or anticoagulant medications. The corresponding odds ratios (OR) and 95% CI were also presented. The influence of medication use on implant outcomes was assessed at both implant and patient level. The statistical analysis was performed using SPSS software, with a significance level set at 0.05 (SPSS v.29.0, IBM, Armonk, NY, USA).

## Results

The patient characteristics in the total population as well as in the antiplatelet and anticoagulant patient groups are shown in Table 1. This study examined 20,842 patients who received a total of 50,333 dental implants between 2011 and 2022. The mean patient age was 57.5 years, with ages ranging from 18 to 94. The majority of patients were female (51.8%), non-Hispanic (91.1%), and white (66.3%). Additionally, 8% of the patients were tobacco users. At the patient level, implant failure rate was 2.7%. Antiplatelet use was reported by 1,985 individuals (9.5%), while 380 patients were users of anticoagulants (1.8%). The univariate analysis on the association between patient characteristics and medication intake demonstrated that age, gender, ethnicity, race, and tobacco use were significantly associated with the use of antiplatelets and anticoagulants. The implant treatment outcome was not significantly associated with the use of any of these two types of medications. The multivariate analysis showed that Asians (OR: 1.90, 95% CI: 1.19–3.03, *p* = 0.01), African-Americans (OR: 1.83, 95% CI: 1.16–2.88, *p* = 0.01), American Indians or Alaskan Natives (OR: 5.57, 95% CI: 2.58–12.04, *p* < 0.001), and White individuals (OR: 3.26, 95% CI: 2.25–4.71, *p* < 0.001) exhibited significantly higher risk of taking antiplatelets when compared to Hispanics or Latinos. Similarly, males (OR: 1.51, 95% CI: 1.36–1.69, *p* < 0.001) and smokers (OR: 2.29, 95% CI: 1.98–2.64, *p* < 0.001) had significantly higher odds of being antiplatelet users when compared to females and non-smokers, respectively. Significantly higher risk of taking anticoagulants was found for males (OR: 1.25, 95% CI: 1.01–1.55, *p* = 0.04) and smokers (OR: 1.59, 95% CI: 1.19–2.14, *p* < 0.001) compared to females and non0smokers, respectively.

**Table 1:** Patient characteristics in the total population as well as in the antiplatelet and anticoagulant patient groups

Patient characteristics		Total population *N* = 20,842 (100%)	Antiplatelets			Anticoagulants		
			Yes *N* = 1,985 (9.5%)	No *N* = 18,857 (90.5%)	*p*-value	Yes *N* = 380 (1.8%)	No *N* = 20,462 (98.2%)	*p*-value
AGE (mean (SD)) [range]		57.50 (14.27) [18–94]	67,17 (9.23) [18–93]	56.49 (14.33) [18–94]	< 0.001	69.60 (9.37) [30–93]	57.28 (14.25) [18–94]	< 0.001
GENDER (%)	Female	10,798 (51.8)	776 (39.1)	10,022 (53.2)	< 0.001	144 (37.9)	10,654 (52.1)	< 0.001
	Male	10,041 (48.2)	1,208 (60.9)	8,833 (46.8)		236 (62.1)	9,805 (47.9)	
ETHNICITY (%)	Non-Hispanic	18,894 (91.1)	1,906 (96.0)	17,088 (90.6)	< 0.001	363 (95.5)	18,631 (91.1)	0.003
	Hispanic	1,294 (6.2)	74 (3.7)	1,220 (6.5)		16 (4.2)	1,278 (6.2)	
	Other	554 (2.7)	5 (0.3)	549 (2.9)		1 (0.3)	553 (2.7)	
RACE (%)	White	9,900 (66.3)	1,419 (89.8)	8,481 (63.5)	< 0.001	283 (92.5)	9,617 (65.7)	< 0.001
	Asian	935 (6.3)	47 (3.0)	888 (6.6)		3 (1.0)	932 (6.4)	
	African American	962 (6.4)	56 (3.5)	906 (6.8)		10 (3.3)	952 (6.5)	
	Hispanic or Latino	1,173 (7.9)	31 (2.0)	1,142 (8.6)		7 (2.3)	1,166 (8.0)	
	Pacific Islander	65 (0.4)	0 (0.0)	65 (0.5)		0 (0.0)	65 (0.4)	
	American Indian or Alaskan Native	59 (0.4)	11 (0.7)	48 (0.4)		1 (0.3)	58 (0.4)	
	Other	1,843 (12.3)	17 (1.1)	1,826 (13.7)		2 (0.7)	1,841 (12.6)	
TOBACCO USE (%)		1,673 (8.0)	177 (19.0)	1,296 (6.9)	< 0.001	73 (19.2)	1,600 (7.8)	< 0.001
Treatment outcome	Survived	20,274 (97.3)	1,928 (97.1)	18,346 (97.3)	0.67	368 (96.8)	19,906 (97.3)	0.53
	Failed	568 (2.7)	57 (2.9)	511 (2.7)		12 (3.2)	556 (2.7)	

In the present investigation, the majority of the included anticoagulant patients were on warfarin (45.9%) and apixaban (31.5%). Rivaroxaban was reported by the 21% of the anticoagulant users, while 1.6% were users of dabigatran. In addition, the majority of the included antiplatelet patients were on aspirin (87.5%) followed by clopidogrel (11.8%), tricagrelor (0.6%), and prasugrel (0.1%). At the implant level, this study evaluated 50,333 dental implants over a mean follow-up period of 83.86 ± 57.57 months (minimum: 0 and maximum: 367 months). Out of these, 725 implants failed, resulting in a failure rate of 1.4%. Most implants were placed in the maxilla (53.5%) and in the posterior region (71.8%). At the implant level, out of the 4,770 implants placed in patients taking antiplatelets, 76 (1.6%) failed, whereas 649 failures occurred in those who did not report antiplatelet use (649 from a total of 45,563) leading to a failure rate of 1.4%. No significant differences were detected in regard to implant failure rate between users and non-users of antiplatelet medications at the implant level (*p* = 0.34). With respect to the anticoagulant group, a failure rate of 1.3% was found in the group of patients reported to be taking anticoagulants, while 1.4% was the respective implant failure rate in non-users. This results in a non-significant difference between the two groups (*p* = 0.89).

**Fig. 1:**
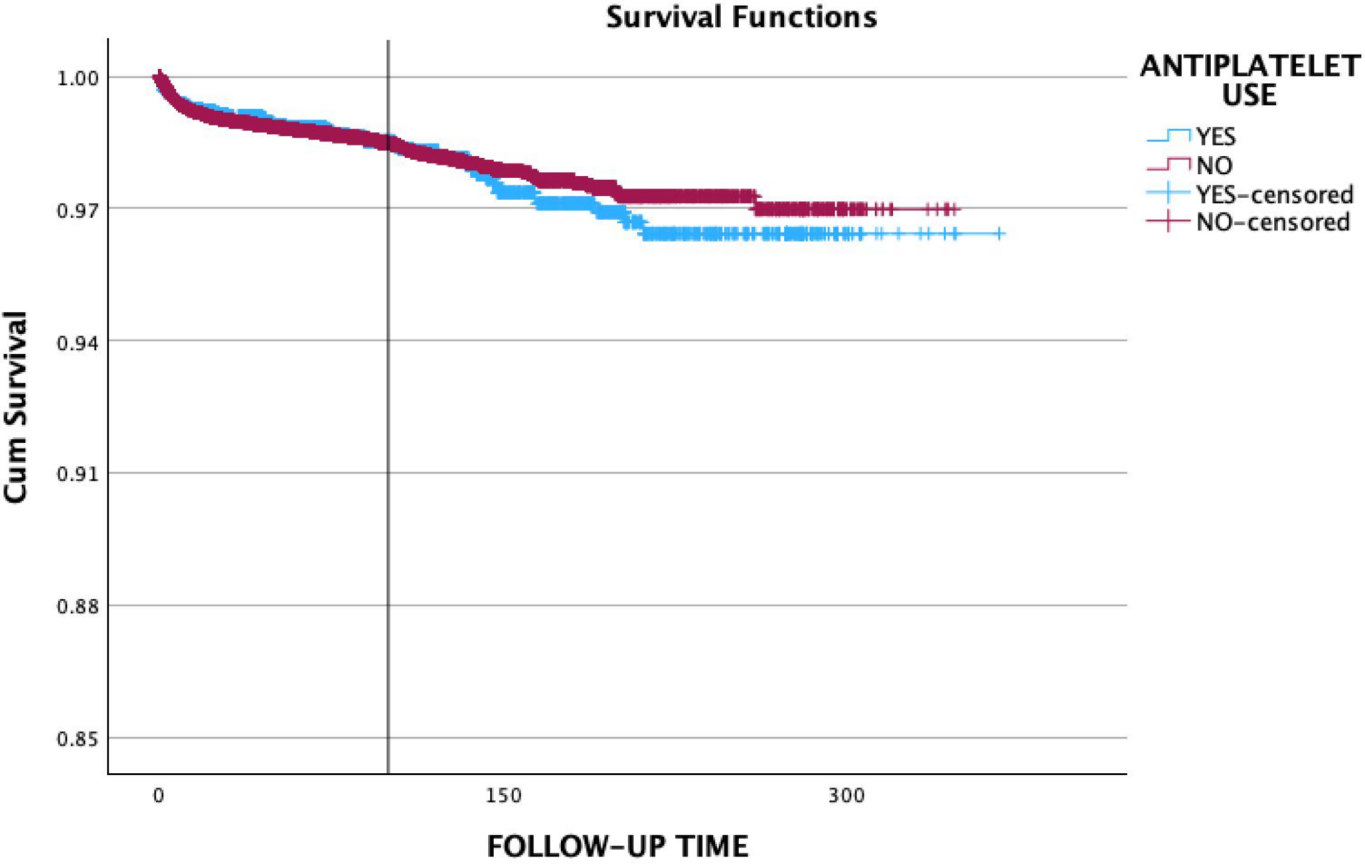
The Kaplan-Meier curves comparing the cumulative survival of implants placed in antiplatelet users compared to non-users

**Fig. 2:**
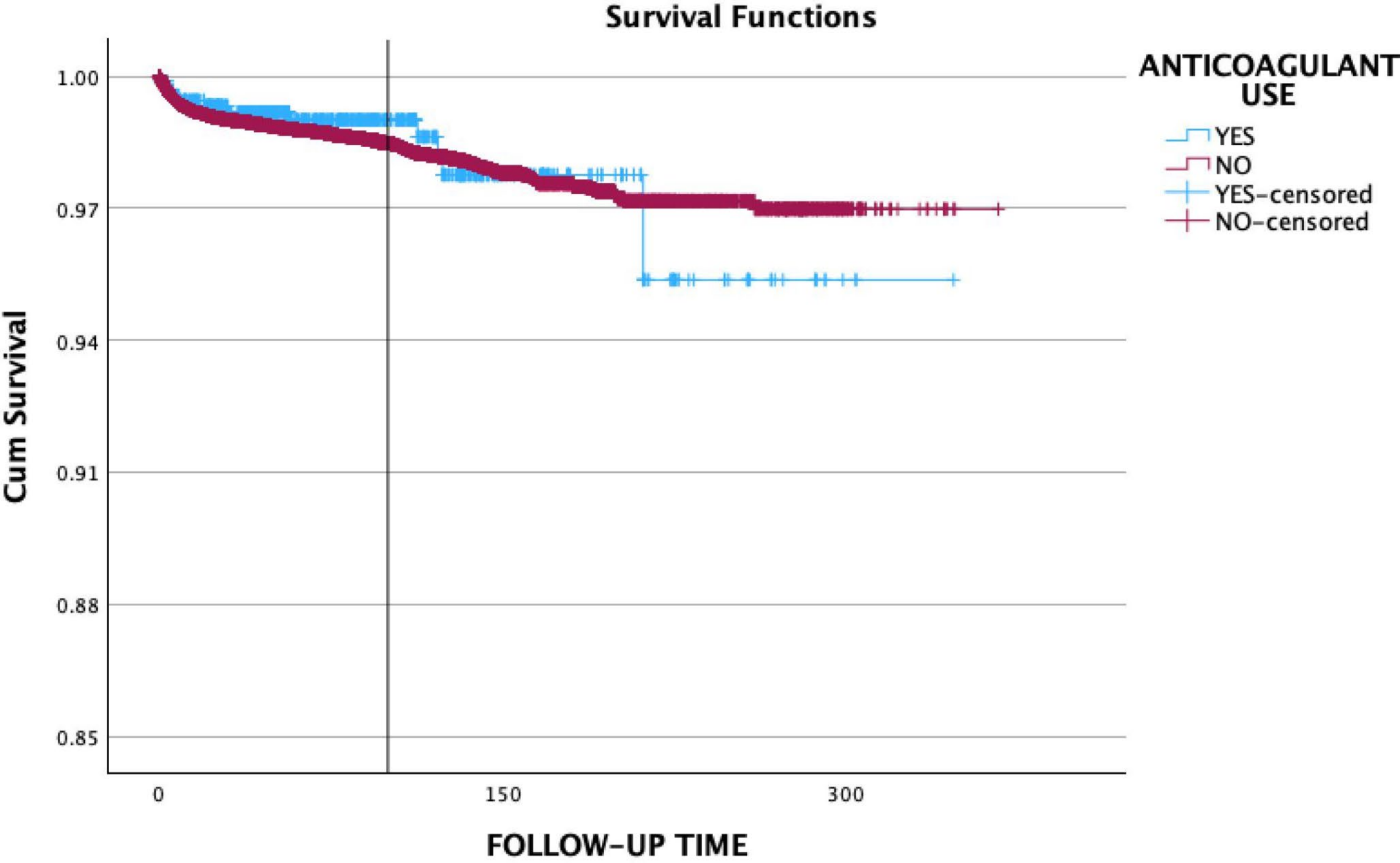
The Kaplan-Meier curves comparing the cumulative survival of implants placed in anticoagulant users compared to non-users

The Kaplan-Meier curves comparing the cumulative survival of implants placed in antiplatelet and anticoagulant users compared to non-users are shown in Figs. 1 and 2, respectively. The mean survival time for implants placed in antiplatelet users was 357.31 as compared to 339.60 months in non-users (*p* = 0.72). The implant survival rate for antiplatelet users was estimated to 98.4% as compared to 98.6% in non-users. Implants placed in anticoagulant users demonstrated a mean survival time of 337.73 months when compared to 358.47 months in the non-users (*p* = 0.70). The implant survival rate for anticoagulant users was 98.7% compared to 98.6% in the non-user group. It is important to interpret the later portion of the Kaplan-Meier curves with caution, particularly beyond 200 months, where the number of patients at risk decreases substantially. This can lead to increased variability and potentially less reliable estimates of survival probabilities in the long term.

## Discussion

This retrospective study used the BigMouth Dental Data Repository to investigate the long-term relationship between antiplatelet and anticoagulant medications with the risk of dental implant failure. The study analyzed 50,333 dental implants in 20,842 patients over 12 years and found an implant failure rate of 1.4% at the implant level and 2.7% at the patient level. In addition, specific racial groups including Asians, African-Americans, American Indians or Alaskan Natives, and White individuals were significantly more likely to receive antiplatelet medications than Hispanics or Latinos. Males and smokers exhibited significantly higher odds of being antiplatelet and anticoagulant users compared to females and non-smokers, respectively. When the implant survival rates between antiplatelet and anticoagulant users were compared to non-users, no significant differences were observed.

Oral anticoagulants are widely used to manage thromboembolic events including thromboembolism in atrial fibrillation, treatment of venous thromboembolism, cerebrovascular accidents, ischemic heart disease, myocardial infarction, bypass surgery, and prosthetic heart valve placement [32]. VKAs have been the traditional choice, but DOAC offer a more convenient option with targeted action, fixed dosing, and no need for routine monitoring [33, 34]. DOACs include factor Xa inhibitors (rivaroxaban, apixaban, edoxaban) and the thrombin inhibitor dabigatran [35]. Platelets are small anucleate cells that are crucial in controlling hemostasis and thrombosis [36]. Due to their role in thrombosis, they are a key target for medications used to treat thrombotic disorders. The most commonly used antiplatelet medications include aspirin, medications that block the P2Y12 receptor on platelets, and inhibitors of the GPIIb/IIIa protein, which is important for platelet aggregation [37–39]. Acetylsalicylic acid (aspirin) is rapidly absorbed and works by inhibiting thromboxane synthesis, reducing platelet activation and aggregation [40, 41]. Clopidogrel, on the other hand, acts as an ADP receptor blocker, altering platelet shape and hindering their aggregation [42]. Dual antiplatelet therapy (DAPT), combining aspirin with either clopidogrel or another P2Y12 inhibitor, is often used in cardiovascular patients to prevent further heart complications or issues after stent placement [43].

To the best of our knowledge this is the first large-scale study comparing the survival rates of implants placed in antiplatelet and anticoagulant users and non-users long-term. Studies have evaluated the effect of such medications on early implant failures [44, 45]. A recent study examined the impact of antiplatelet and anticoagulant medications on dental implant outcomes after sinus floor augmentation [44]. The researchers included 110 patients with 305 implants, and they showed no increased risk of early implant failure or bleeding complications in patients on these medications [44]. However, smoking, lower initial bone height, and staged implant placement were identified as risk factors for implant failure [44]. Another study by Chaushu et al. examined retrospectively the effect of anticoagulants on early implant failure and included 687 patients who had 2,971 implants placed in a hospital [45]. In that study early implant failure was defined as an implant failure that occurred within the first 12 months from loading. Implants placed in patients on anticoagulants exhibited increased odds of early implant failure (OR: 2.64) and implants in people over 80 years old or with certain health conditions had lower odds of early implant failure [45].

A 6-year retrospective cohort study that examined factors affecting the long-term success of dental implants analyzed a sample of 297 implants over 76 months, focusing on failure of osseointegration and survival of implants with an internal connection and machined collar [46]. The authors reported a high survival rate, but also identified gender, smoking, and anticoagulant use as risk factors [46]. The results showed a 4.4% reduction in survival rate for patients taking these medications, with an odds ratio of failure at 28.2. The study concluded that anticoagulant use significantly increases the risk of implant failure [46]. Chrcanovic et al. conducted a study investigating the impact of various systemic risk factors, including antithrombotics (anticoagulants and antiplatelet medications), on early implant failure [47]. Their findings revealed no significant association between the use of these medications and early implant failure [47]. Therefore, the available evidence is contradictory and large-scale long-term studies are needed to validate and examine the impact of anticoagulants on the osseointegration. The present investigation that included records of 50,333 implants inserted in 20,842 patients showed that no significant association exists between anticoagulant medications and implant failure. The majority of the included anticoagulant patients were on warfarin (45.9%) and apixaban (31.5%). Rivaroxaban was reported by the 21% of the anticoagulant users, while 1.6% were users of dabigatran.

The effect of antiplatelet use on implant treatment outcome has been examined only by a recent retrospective cohort study which focused their analysis on early implant failures [44]. This study reported an implant failure rate of 4.65% at the implant level and 10% at the patient level. The authors concluded that implant failure was not significantly associated with the use of antiplatelets [44], which is in agreement with our findings for the long-term implant survival. A study that used a rabbit model to investigate if continuing clopidogrel during and after surgery negatively impacts bone healing included 16 rabbits and the authors divided them into two groups: one receiving clopidogrel and the other a placebo [24]. Both groups underwent surgery to create circular calvarial defects and were monitored for 6 weeks. The results showed no adverse effects from clopidogrel; in fact, the clopidogrel group had significantly better bone regeneration and defect bridging based on radiographic and microscopic examination [24]. A recent animal study that examined the effect of clopidogrel on bone healing around titanium dental implants included 16 rabbits that were on clopidogrel and another 16 rabbits serving as controls [48]. The clopidogrel group showed significantly better bone-to-implant contact, bone density, and trabecular thickness compared to the control group which suggests that clopidogrel does not hinder, but rather enhances, osseointegration [48].

Another study examined how dual antiplatelet medications impact bone regeneration around dental implants in rats [49]. The rats were divided into groups receiving different combinations of antiplatelet drugs including acetylsalicylic acid, acetylsalicylic acid + clopidogrel, clopidogrel, acetylsalicylic acid + prasugrel, ticagrelor, acetylsalicylic acid + ticagrelor, or no treatment (control). After 8 weeks, the researchers found no significant differences between the groups in terms of bone regeneration which demonstrates that antiplatelet therapy may not negatively affect bone healing around implants [49]. In addition, an animal study examined how long-term low-dose aspirin affects the implant osteointegration [50]. Rats were given either aspirin or a placebo for different durations before and after receiving implants. Analysis of bone-to-implant contact and bone area between threads showed that short-term aspirin use (7 days) slightly reduced bone formation around the implants compared to the control group and long-term aspirin use [50]. However, this effect disappeared after 28 days, suggesting that low-dose aspirin may only have a temporary impact on early bone healing around implants [50]. In the present investigation, the majority of the included antiplatelet patients were on aspirin (87.5%) followed by clopidogrel (11.8%), tricagrelor (0.6%), and prasugrel (0.1%).

This study has limitations due to its retrospective nature, its focus on university dental clinic patients (who may have more complex medical conditions than the general population), and the involvement of multiple surgeons with varying preoperative practices as well as variations in implant placement techniques. However, standardized implant protocols and the use of high-quality implants across all centers reduce this concern. It is important to acknowledge the potential for confounding in our study due to the imbalance in sample sizes and baseline characteristics between the antithrombotic therapy groups and the non-antithrombotic group. The significantly larger size of the non-antithrombotic group, coupled with differences in factors like age, gender, and tobacco use, could introduce bias and affect the interpretation of our findings. Ideally, matching techniques would be employed to create comparable groups and minimize confounding. However, due to the limitations of our retrospective data and the availability of relevant variables, exact matching was not feasible. Future prospective studies with more balanced groups and comprehensive data collection are needed to confirm our findings and further explore the relationship between antithrombotic therapy and implant outcomes. A key limitation of our study is the lack of granular data on certain patient characteristics and treatment variables. Specifically, our dataset did not consistently capture information on the dosage and duration of antithrombotic medication use. This missing information prevents us from exploring potential dose-dependent or time-dependent effects of these medications on implant outcomes. While the data did not include specific details on medication dosage and duration, the analysis was restricted to patients who remained on their prescribed antithrombotic therapy throughout the entire follow-up period. Additionally, we lacked detailed data on certain potential confounders, such as specific systemic diseases, concomitant medications, and other factors like bone quality, implant placement technique and periodontal status and maintenance care. These factors could influence implant survival and may have contributed to residual confounding in our analysis.

Future prospective studies with comprehensive follow-up data are needed to confirm or refute a causal link between anticoagulant and antiplatelet use and implant failure, taking into account the duration and dosage of medication and other potential confounding factors. Future prospective studies with standardized data collection protocols should include detailed documentation of treatment indications to allow for a more comprehensive analysis of their impact on implant outcomes in patients receiving antithrombotic therapy. Strengths of this study include its large dataset from multiple universities and a remarkably long follow-up period, enhancing the robustness of its findings and offering valuable long-term perspectives on implant survival.

## Conclusions

Within the limitations of this study, it appears that the use of anticoagulant and antiplatelet medications does not affect the risk of implant failure. Both anticoagulant and antiplatelet users and non-users exhibit similar high implant survival rates. Further well-designed prospective studies are necessary to confirm these findings and explore other potential risk factors.

## Data Availability

No datasets were generated or analysed during the current study.
